# Validation of Candidate Serum Ovarian Cancer Biomarkers for Early Detection

**Published:** 2007-10-16

**Authors:** Feng Su, Jennifer Lang, Ashutosh Kumar, Carey Ng, Brian Hsieh, Marc A. Suchard, Srinivasa T. Reddy, Robin Farias-Eisner

**Affiliations:** 1 Department of Obstetrics and Gynecology, University of California, Los Angeles, CA 90095, U.S.A; 2 Pathway Diognostics, Inc. Malibu, CA 90265, U.S.A; 3 Biomathematics and Human Genetics; 4 Biostatistics, School of Public Health; 5 Medicine, and; 6 Departments of Molecular and Medical Pharmacology, University of California, Los Angeles, CA 90095, U.S.A

**Keywords:** Ovarian cancer, Serum biomarker, Serous, Mucinous

## Abstract

**Objective:**

We have previously analyzed protein profiles using Surface Enhanced Laser Desorption and Ionization Time-Of-Flight Mass Spectroscopy (SELDI-TOF-MS) [Kozak et al. 2003, *Proc. Natl. Acad. Sci. U.S.A.* 100:12343–8] and identified 3 differentially expressed serum proteins for the diagnosis of ovarian cancer (OC) [Kozak et al. 2005, *Proteomics*, 5:4589–96], namely, apolipoprotein A-I (apoA-I), transthyretin (TTR) and transferin (TF). The objective of the present study is to determine the efficacy of the three OC biomarkers for the detection of early stage (ES) OC, in direct comparison to CA125.

**Methods:**

The levels of CA125, apoA-I, TTR and TF were measured in 392 serum samples [82 women with normal ovaries (N), 24 women with benign ovarian tumors (B), 85 women with ovarian tumors of low malignant potential (LMP), 126 women with early stage ovarian cancer (ESOC), and 75 women with late stage ovarian cancer (LSOC)], obtained through the GOG and Cooperative Human Tissue Network. Following statistical analysis, multivariate regression models were built to evaluate the utility of the three OC markers in early detection.

**Results:**

Multiple logistic regression models (MLRM) utilizing all biomarker values (CA125, TTR, TF and apoA-I) from all histological subtypes (serous, mucinous, and endometrioid adenocarcinoma) distinguished normal samples from LMP with 91% sensitivity (specificity 92%), and normal samples from ESOC with a sensitivity of 89% (specificity 92%). MLRM, utilizing values of all four markers from only the *mucinous* histological subtype showed that collectively, CA125, TTR, TF and apoA-I, were able to distinguish normal samples from *mucinous* LMP with 90% sensitivity, and further distinguished normal samples from early stage *mucinous* ovarian cancer with a sensitivity of 95%. In contrast, in serum samples from patients with *mucinous* tumors, CA125 alone was able to distinguish normal samples from LMP and early stage ovarian cancer with a sensitivity of only 46% and 47%, respectively. Furthermore, collectively, apoA-I, TTR and TF (excluding CA-125) distinguished i) normal samples from samples representing all histopathologic subtypes of LMP, with a sensitivity of 73%, ii) normal samples from ESOC with a sensitivity of 84% and iii) normal samples from LSOC with a sensitivity of 97%. More strikingly, the sensitivity in distinguishing normal versus *mucinous* ESOC, utilizing apoA-I, TF and TTR (CA-125 excluded), was 95% (specificity 86%; AUC 95%).

**Conclusions:**

These results suggest that the biomarker panel consisting of apoA-I, TTR and TF may significantly improve early detection of OC.

## Introduction

Ovarian cancer has the highest mortality rate of all the gynecologic malignancies worldwide. With no adequate screening tests, early diagnosis—the most significant prognostic factor—continues to elude the clinician. Presently, over 85% of patients with ovarian cancer are diagnosed with Stage III or IV disease [1].

Serum cancer antigen 125 (CA125), a high molecular weight glycoprotein, is currently the best clinical marker for papillary serous adenocarcinoma of the ovary in the postmenopausal age group. However it is a consistently poor diagnostic tumor biomarker in premenopausal women, non-serous histologies, and early stage diseases. Only 50%–60% of women with early stage ovarian cancer will demonstrate elevated serum levels of CA125 [2]. Falsely elevated levels are common in a number of benign conditions such as pregnancy, uterine fibroids, or intra-abdominal infections and other intraperitoneal pathology [3]. The identification of more sensitive and specific biomarkers for the early detection of ovarian cancer would clearly be immediately beneficial.

Proteomic-based approaches have been utilized in an attempt to detect early-stage ovarian cancer patients, and monitor biologic responses to therapy [4], [5]. Serum protein profiling at different stages in disease progression, or along the course of therapy, offers a new paradigm for detecting and treating ovarian cancer [6–10]. We have previously analyzed protein profiles using Surface Enhanced Laser Desorption and Ionization Time-Of-Flight Mass Spectroscopy (SELDI-TOF-MS) and identified 3 differentially expressed serum proteins for the detection of ovarian cancer [6], [7]. These were apoA-I, TTR, and TF. In the present study, we analyzed an additional 392 serum samples from patients obtained through the GOG and Cooperative Human Tissue Network for the levels of markers that included CA125, in addition to the previously described markers.

## Materials and Methods

Serum samples were obtained through the Gynecological Oncology Group (GOG) and Cooperative Human Tissue Network. Samples were collected preoperatively following the standard GOG protocol (GOG 199 protocol) from patients with benign, borderline and malignant ovarian tumors. The 392 serum samples utilized in the present study included 82 women with normal ovaries (N), 24 women with benign ovarian tumors (B), 85 women with ovarian tumors of low malignant potential (LMP), 126 women with early stage ovarian cancer (ESOC), and 75 women with late stage ovarian cancer (LSOC). The age and pathology distribution of the samples are provided in Table 1.

The levels of each individual protein marker (CA125, apoA-I, TTR, TF) were measured on all serum samples. The Immulite 1000 was used to measure CA125 level by using chemiluminescence technology and the Hitachi 912 was used to measure apoA-I, TTR and TF levels based on immunoturbimetry technology. The reagents were purchased from Diagnostics Product Corporation and Roche. A separate dataset was compiled for external-validation purposes from serum collected from patients with breast cancer, colon cancer and atherosclerosis.

Statistical analysis of the levels of each of the individual markers (apoA-I, TTR, TF, and CA125) was performed using the Kruskal-Wallis non-parametric rank sum test and Mann-Whitney U tests to compare marker levels across ovarian cancer stage. Multivariate logistic regression models (MLRM) were built to predict N vs. low malignant potential (LMP) and N vs. ESOC and LSOC. Model prediction ‘cut-points’ were also determined by maximizing specificity and sensitivity with equal weight. We then compared MLRM sensitivity, specificity and area under the receiver operator curve (AUC). AUC is a cut-point independent measure of predictive value.

Age-matched (51.5 ± 7.5) sera from a separate dataset that included normals, patients with early stage ovarian cancer, breast and colon cancers, and atherosclerosis were then standardized based on the normals in each dataset, assuming a scalar multiplier for each type of measurement (CA125, apoA-I, TF, and TTR). To compute the standardization, multipliers and perform multivariate statistical tests, extreme outliers were removed using a standard outlier detection procedure [10], [11]. The multivariate MANOVA and MLRM prediction was then employed to demonstrate marker level differences between these external validation patients and the ovarian cancer patients. To determine statistical significance, we assumed a 5% Type I Error rate and did not control for multiple comparisons. All tests were performed using the R statistical package.

## Results

Prior to analyzing for the new biomarker panel, the serum was first tested for CA125 levels, which is the current gold standard biomarker for ovarian cancer. Although CA125 had 99% specificity for all the samples tested, the sensitivity was only 62% in distinguishing LMP from normal subjects, and 76% in distinguishing ESOC from normal subjects (Table 2). Moreover, in serum samples from patients with *mucinous* tumors, CA125 was able to distinguish normal samples from LMP and ESOC with a sensitivity of only 46% and 47%, respectively (Table 2).

**Figure 1:**
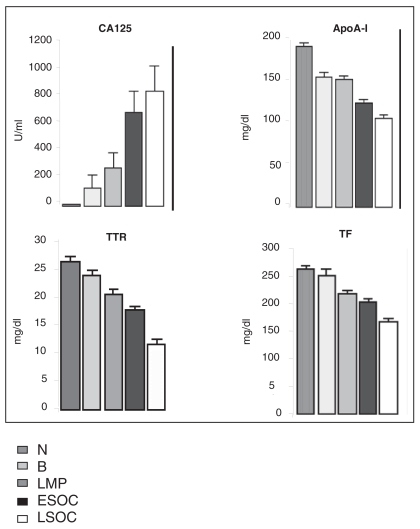
The comparisons of differential protein expression in serum across subgroups. Plotted values are 1 standard error. Using Kruskal-Wallis ranks sum test, expression for each protein significantly differs across subgroups from normal, all p-values < 0.001.

The expression of each protein from the biomarker panel is significantly different across subgroups compared to normal (Fig. 1). Multiple logistic regression models (MLRM) were constructed utilizing all biomarker values (CA125, TTR, TF and apoA-I) from all histological subtypes (serous, *mucinous*, and endometrioid adenocarcinoma). Collectively, the four markers (CA125, TTR, TF and apoA-I) distinguished normal samples from LMP with 91% sensitivity (specificity 92%; AUC 97%), normal samples from ESOC with a sensitivity of 89% (specificity 97%; AUC 98%); and normal samples from LSOC with a sensitivity of 97% (specificity 99%; AUC 99%) (Table 3).

Interestingly, when a similar logistic regression analysis was performed evaluating only the *mucinous* histology subgroup, the results suggested greater sensitivity and specificity in the ability of the biomarkers to distinguish serum from normal subjects versus patients with ESOC (Table 3). MLRM, utilizing all biomarker values (CA125, TTR, TF and apoA-I), from only the *mucinous* histological subtype showed that collectively, CA125, TTR, TF and apoA-I were able to distinguish normal samples from *mucinous* LMP with 90% sensitivity (specificity 91%; AUC 96%). In contrast, these four biomarkers when used together, distinguished serum samples from normal subjects versus patients with early stage *mucinous* ovarian cancer with a sensitivity of 95% (specificity 92%; AUC 97%) (Table 3).

We next assessed the utility of the new biomarker panel consisting of TTR, TF and apoA-I for the early detection of OC. MLRM was constructed using the values for apo-A1, TF and TTR (CA125 excluded, Table 4). Collectively, apoA-I, TTR and TF distinguished i) normal samples from samples representing all histopathologic subtypes of LMP, with a sensitivity of 73% (specificity 83%; AUC 81%), ii) normal samples from samples with ESOC with a sensitivity of 84% (specificity 85%; AUC 90%), and iii) normal samples from LSOC with a sensitivity of 97% (specificity 86%; AUC 96%). More strikingly, the sensitivity in distinguishing normal versus *mucinous* early stage ovarian cancer, utilizing apoA-1, TF and TTR (CA125 excluded), was 95% (specificity 86%; AUC 95%) (Table 4).

**Figure 2:**
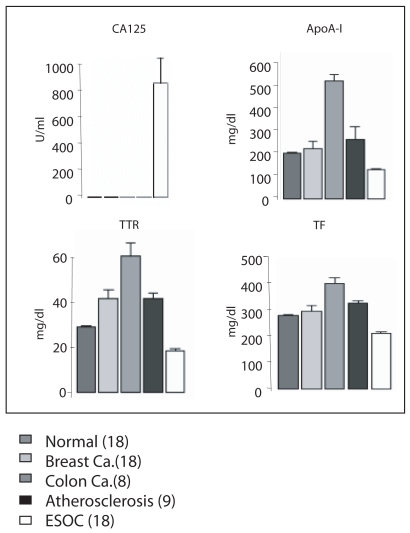
The standardized comparisons of differential protein expression in serum across different diseases. Plotted values are 1 standard error. Using a robust MANOVA analysis, CA125, apoA-I, TTR and TF are significantly differ only in early stage ovarian cancer from normal samples, all p-values < 0.0001.

**Figure 3:**
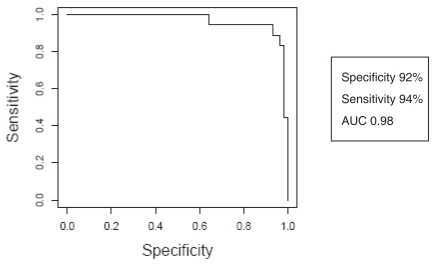
Receiver operating characteristic curve of multivariate predictive model using apoA-I, TTR and TF.

To further validate the disease-specificity of the three biomarkers, we examined serum levels for apoA-I, TTR and TF in 71 additional subjects that included normal (18), breast cancer (18), colon cancer (8), atherosclerosis (9), and early stage OC (18) (Fig. 2). Multivariate comparison of apoA-I, TF and TTR demonstrate notable differences between diseases (Fig. 2). Using the MLRMs constructed to make predictions on these independent data resulted in the ROC curve (Fig. 3), and demonstrated a specificity of 92%, sensitivity of 94% and AUC of 0.98.

## Discussion

The majority of patients with ovarian cancer are diagnosed with Stage III or IV disease. Unfortunately, there are no adequate screening tests for the early detection of ovarian cancer and as a result, the diagnosis of ovarian cancer eludes the clinician. Not surprisingly, ovarian cancer is associated with the highest mortality rate among gynecologic malignancies. [1].

Serum cancer antigen 125 (CA125), a high molecular weight glycoprotein, is currently the best clinical marker for papillary serous adenocarcinoma of the ovary in the postmenopausal age group. However it is a consistently poor diagnostic tumor biomarker in premenopausal women, non-serous histologies, and early stage diseases. Only 50%–60% of women with early stage ovarian cancer will demonstrate elevated serum levels of CA125 [2]. Falsely elevated levels are common in a number of benign conditions such as pregnancy, uterine fibroids, or intra-abdominal infections and other intraperitoneal pathology [3]. The identification of more sensitive and specific biomarkers for the early detection of ovarian cancer would clearly be immediately beneficial.

Since CA125 is the gold standard biomarker for ovarian cancer, we measured CA125 levels in all the study samples. CA125 levels alone distinguished N from LMP with a sensitivity of 62% and N from ESOC with a sensitivity of 76% (Table 2). Furthermore, when the *mucinous* subsets were analyzed, CA125 levels distinguished N from LMP and ESOC with a sensitivity of 46% and 47% respectively (Table 2). These numbers are in agreement with previously reported data for CA125 [12]. As one of the goals of this study was to test the efficacy of the three biomarkers we recently identified for the detection of OC, we examined whether the three markers, apoA-I, TTR and TF could improve upon the CA125 based measurements. Using all the four markers (apoA-I, TTR, TF and CA125) and all of the 392 samples we analyzed for this study, we observed a 29% improvement in sensitivity for the detection of LMP, and a 13% improvement in sensitivity for the detection of ESOC (Table 3). More importantly, the four markers collectively improved the detection of LMP and ESOC of the *mucinous* subtype by 44% and 48%, respectively, compared to normal subjects (Table 3). These results warrant further studies to evaluate the new biomarkers in the early detection of OC.

Interestingly, there exists a link between OC and each of the three biomarkers, apoA-I, TTR and TF [13], [14], [15]. ApoA-I (28 kDa) is the major protein constituent of high density lipoprotein. Decreased apoA-I levels were previously reported in the serum of patients with both ovarian cancer [13], [14], [15] as well as atherosclerosis [16]. Serum lipid and lipoprotein association with cancer has been reported in numerous studies [17], [18], [19]. The mechanism of this association remains unclear at this time, however it has been proposed to be associated with free radical-mediated damage to cellular biomembranes resulting in lipid peroxidation. Malondiadlehyde (MDA) is a byproduct of lipid degradation. MDA-DNA adducts appear to be promutagenic, inducing mutations in oncogenes and tumor suppressor genes seen in human tumors [20]. TTR (13.9 kDa) is a secreted protein that functions as a binding protein to transport serum thyroxine, tri-iodothyronine and retinol (vitamin A). TTR levels have been reported to be inversely correlated to tumor volume in ovarian cancer [21]. Immunohistochemistry studies have shown levels of cellular retinal binding proteins to be decreased in ovarian cancers [22]. TF (79 kDa) is an iron binding transport protein, responsible for transporting iron from sites of iron absorption and heme degradation to areas of storage and utilization [23], [24]. TF has been previously reported to be decreased in serum of patients with ovarian cancer [25]. All of these molecules have been shown to play an important role in oxidative stress, for which there is myriad data linking to carcinogenesis [26], [27], [28], [29], [30].

In order to evaluate the direct efficacy of the three biomarkers, apoA-I, TTR, and TF, in detecting OC, we reevaluated the specificity, sensitivity and AUC values without CA125 levels. We noted an improvement of 11% in sensitivity for the detection of LMP (compared to CA125 alone, Table 2 and Table 4) when samples from all histopathologic subtypes were analyzed, and an improvement of 14% for the detection of LSOC (compared to CA125 alone, Table 2 and Table 4). Interestingly, we noted an improvement of 34% in sensitivity for the detection of LMP (compared to CA125 alone, Table 3 and Table 4) when mucinous histopathologic subtypes alone were analyzed, and an improvement of 48% for the detection of LSOC (compared to CA125 alone, Table 3 and Table 4). These data further attest to the clear improvement (over CA125) of the new panel of biomarkers for the early detection of OC. These results further build on the work previously reported by Zhang et al. (2004), also utilizing SELDI-TOF-MS with similar methodology, in which a panel of biomarkers including apolipoprotein A1, and a truncated form of transthyretin (identified from the m/z 12828 peak, corresponding to a 12.9 kDa protein fragment), in combination with a cleavage factor of inter-a-trypsin inhibitor heavy chain H4 and CA125 saw a more modestly improved sensitivity (9%) for the detection of stage I/II ovarian cancer over CA125 alone [31]. It is interesting that our work identified the m/z 13797 peak corresponding to the 13.9 kDa marker, which we identified as the complete TTR protein product to be down-regulated in early ovarian cancer.

More recently, to evaluate these markers in an independent study population, postdiagnostic/pre-treatment serum samples were studied as part of the National Cancer Institute Immunodiagnostic Serum Bank; levels of various posttranslationally forms of transthyretin and apolipoprotein A1 were measured in addition to CA125. The mean levels of five of the six forms of transthyretin were significantly lower in cases than in controls. The specificity of a model including transthyretin and apolipoprotein A1 alone was high, 96.5%, but sensitivity was low, 52.4%. A class prediction algorithm using all seven markers, CA125, and age maintained high specificity, 94.3%, but still relatively low sensitivity, 78.6% [32]. It is interesting that our work identified the m/z 13797 peak corresponding to the 13.9 kDa marker, which we identified as the complete TTR protein product to be differentially expressed as down-regulated in early ovarian cancer.

Finally, our data also suggest that apoA-I, TTR, and TF, when analyzed collectively, are unique to ovarian cancer (Fig. 2) and thus provide for the first time a disease-specific multiple marker panel for the early detection of OC. In conclusion, we have shown that ApoA-I, TF, and TTR, in combination with CA125 in a multivariate predictive model, have the potential to improve the specificity and sensitivity for the early detection of ovarian cancer over CA125 alone, particularly for the mucinous histopathologic subtype. Further elucidation of the mechanisms and pathways by which ApoA-I, TF, and TTR, participate in the development of OC will not only be important for early detection but also ultimately provide targets for therapeutic intervention of OC.

## Article Précis

Three serum biomarkers, ApolipoproteinA-I, Transthyretin and Transferrin, combine with CA125 can be used to significantly improve detection of early stage ovarian cancer over CA125 alone.

## Figures and Tables

**Table 1:** Clinical characteristics and age distribution of 392 study samples.

		Age				Pathology			
Diagnostic group	Number	Mean	SD	Median	Clear cell	Endometrioid	Mucinous	Serous	Other
N	82	42.5	10.7	43					
B	24	53.0	18.6	50			10	1	13
LMP	85	50.5	14.9	50		1	41	42	1
ES	126	56.1	13.3	54	15	46	19	25	21
LS	75	59.1	12.5	59	6	10	1	42	16

**Table 2:** Specificity, sensitivity and AUC values derived from CA125 levels ≥35 units/ml for distinguishing normal subjects (n = 82) from LMP, ESOC, LSOC, *mucinous* LMP and *mucinous* ESOC.

Groups	Specificity	Sensitivity	AUC
LMP (all subtypes, n = 85)	0.99	0.62	0.80
ESOC (all subtypes, n = 126)	0.99	0.76	0.87
LSOC (all subtypes, n = 75)	0.99	0.95	0.97
LMP (mucinous, n = 41)	0.99	0.46	0.73
ESOC (mucinous, n = 19)	0.99	0.47	0.73

**Table 3:** Specificity, sensitivity and AUC values derived from all covariates (apoA-I, TTR, TF and CA125) levels for distinguishing normal subjects (n = 82) from LMP, ESOC, LSOC, *mucinous* LMP and *mucinous* ESOC.

Groups	Specificity	Sensitivity	AUC
N vs. LMP (all subtypes, n = 85)	0.92	0.91	0.97
N vs. ES (all subtypes, n = 126)	0.97	0.89	0.98
N vs. LS (all subtypes, n = 75)	0.99	0.97	0.99
N vs. LMP (mucinous, n = 41)	0.91	0.90	0.96
N vs. ES (mucinous, n = 19)	0.92	0.95	0.97

**Table 4:** Multivariate logistic regression models using apoA-I, TF and TTR (CA-125 excluded), for either all histopathologic subtypes or for mucinous subtype alone from 82 normal samples.

Groups	All histological subtypes				Mucinous subtype			
	n	Specificity	Sensitivity	AUC	n	Specificity	Sensitivity	AUC
N vs. LMP	85	0.83	0.73	0.81	41	0.86	0.80	0.88
N vs. ESOC	126	0.85	0.84	0.90	19	0.86	0.95	0.95
N vs. LSOC	75	0.86	0.97	0.96	--	--	--	--
